# Procalcitonin as an Early Laboratory Marker of Sepsis in Neonates: Variation in Diagnostic Performance and Discrimination Value

**DOI:** 10.21315/mjms2019.26.4.7

**Published:** 2019-08-29

**Authors:** Julia Omar, Salbiah Isa, Tuan Salwani Tuan Ismail, Najib Majdi Yaacob, Noor Azlin Azraini Che Soh

**Affiliations:** 1 Department of Chemical Pathology, School of Medical Sciences, Universiti Sains Malaysia, Kubang Kerian, Kelantan, Malaysia; 2 Life Style Science Cluster, Advanced Medical and Dental Institute, Universiti Sains Malaysia, Bertam, Kepala Batas, Pulau Pinang, Malaysia; 3 Units of Biostatistics and Research Methodology, School of Medical Sciences, Universiti Sains Malaysia, Kubang Kerian, Kelantan, Malaysia; 4 Hospital USM, Universiti Sains Malaysia, Kubang Kerian, Kelantan, Malaysia

**Keywords:** procalcitonin, neonatal sepsis, diagnostic performance, discrimination value

## Abstract

**Background:**

As an early recognition of neonatal sepsis is important for triggering the initiation of treatment, this study was thus designed to assess the diagnostic performance and discrimination value of procalcitonin (PCT) in neonatal sepsis cases.

**Methods:**

This cross-sectional study, which was carried out at the Paediatric Intensive Care Unit of Hospital Universiti Sains Malaysia (HUSM) in Kelantan, Malaysia, had involved 60 neonates admitted for suspected sepsis. Sensitivity, specificity, positive predictive values (PPV), negative predictive values (NPV) and the area under receiver operating characteristics curve (AUC) for PCT were determined at initial presentation (0 h) as well as 12 h and 24 h after presentation in comparison to blood culture as the gold standard.

**Results:**

The study consisted of 27 (45.0%) male and 33 (55.0%) female neonates with a mean (SD) age of 76.8 (48.25) h. At cut-off PCT value of > 2 ng/mL, the sensitivity, specificity, PPV and NPV were 66.7%, 66.7%, 33.3% and 88.9% at 0 h. The respective parameters were 83.3%. 56.3%, 32.3% and 93.1% at 12 h and 83.3%, 52.1%, 30.3% and 92.6% at 24 h. AUC was 71.6%, 76.6% and 71.7% at 0 h, 12 h and 24 h.

**Conclusions:**

Diagnostic performance and discrimination values of PCT for diagnosis of neonatal sepsis varied with time of obtaining the blood samples. The PCT result at 12 h demonstrates the most optimal diagnostic performance and discrimination values.

## Introduction

Neonatal sepsis is the leading cause of neonatal morbidity and mortality, which has become an on-going major global public health challenge (1, 2). For this reason, an early and accurate detection of this condition is not only seen as essential for providing a secondary prevention of neonatal sepsis with the initiation of prompt treatments that reduce the mortality rates and devastating complications among the neonates, but also for preventing the possibilities of antibiotics resistance from the use of unnecessary antibiotic prescriptions (2).

In general, while many developing countries are still facing a sub-standard method in the detection of neonatal sepsis, the non-specific signs and symptoms of this disease had made it even harder for most of the sophisticated hospital settings to arrive at a correct clinical diagnosis (3). Since the use of the currently available laboratory tests such as blood cultures, haematological tests and C-reactive protein (CRP) were found to be limited by their respective laboratory and clinical properties (2), this had therefore prompted the constant search of an ideal sepsis biomarker such as those of cell surface markers, acute phase proteins, cytokines and chemokines (4, 5).

Of all the biomarkers that had been evaluated, many authors had found the procalcitonin (PCT) to be a promising marker for the diagnosis of neonatal sepsis (6). Procalcitonin is a 116 amino acid peptide with an approximate 13kDa molecular weight that is consisted of the N-terminal (N-ProCT), C-terminal (catacalcin) and a middle sequence, which can be transformed to that of a calcitonin (7). Apart from having a higher discriminative ability than the white blood cells count (WBC) (2) in distinguishing a bacterial infection from another inflammatory process (2, 8) the early infection diagnosis, assessment of the degree of microbial invasion, severity of the illness and evaluation of response to antibiotics of the PCT were also found to have been more reliable than that of the CRP (9). As such, the PCT can now be considered as an alternative diagnostic tool to the standard blood cultures, where the latter had long been recognised as the gold standard of neonatal sepsis diagnosis (10).

Although PCT is now being used in many countries because of its rapid and higher diagnostic accuracy for diagnosis of neonatal sepsis, the issues concerning its cut-off level for discriminating neonatal sepsis, the optimal diagnostic values as well as the best time for measuring the PCT in neonatal sepsis are still subjected to debate. As such, this study had not only aimed to evaluate the diagnostic value of the test among the neonates in our clinical setting, but was also designed to determine the best time point(s) of using the three PCT serial measurements since the identification of the best evaluation time can offer better clinical decision for the attending physicians, avoid the discriminant use of the PCT testing and cost-savings to the laboratory.

## Methods

A cross-sectional study had been carried out at the Paediatric Intensive Care Unit of Hospital Universiti Sains Malaysia (HUSM) in Kelantan Malaysia, which had involved 60 neonates that had been admitted with suspected sepsis.

Inclusion criteria included suspected neonatal sepsis due to either preterm ruptured of membrane or prolonged ruptured of membrane, maternal infection, chorioamnionitis, group B streptococcus (GBS) colonisation, or signs of foetal distress during labour. Neonates presented with signs and symptoms that are normally associated with neonatal sepsis such as feeding intolerance, lethargic or tachypnic look, poor perfusion, seizures, respiratory distress, bradycardia, abdominal distention, or vomiting were also included. All neonates whose parents had refused to sign the consent forms had been excluded from this study.

Sample size calculation was made based on a calculation for diagnostic test’s estimation of sensitivity and specificity. A previous study reported 10.4% prevalence of early onset neonatal sepsis (11) and another study reported specificity of PCT for diagnosis of neonatal sepsis as being 79% (12). For an estimation with a 95% level of confidence and ± 10% margin of error, the required number of neonates was 72. During the study period, 80 neonates were assessed for eligibility, and only 60 were eligible. By taking 60 neonates, the margin of error for specificity was ± 10.9%. No sampling method was applied in this study.

The characteristics of the neonates; their respective age, weight to the nearest 0.01 kg, gender, race (Malay or non-Malay), onset of suspected sepsis (less than, equal to, or more than 48 h of life) and the gestational age at birth were assessed during the initial evaluation period (0 h).

The PCT blood samples from the eligible neonates were collected at presentation, prior to the administration of antibiotic therapy (0 h) and again at 12 h and 24 h post-presentation. A positive sepsis would be indicated by values of more than 2 ng/mL from the use of the electrochemiluminescence technique on Cobas e411.

The blood samples for the culture test were collected prior to the antibiotic therapies and subsequently incubated in the BACTEC 9240 blood culture system. The presumptive presence of viable microorganisms would be indicated by the positive readings of the BACTEC instrument.

The SPSS Statistics version 22.0 (SPSS Inc. Chicago, IL, USA) was used to examine the missing data, errors from data entry, the distribution of each numerical variable as well as the frequency and percentage of the categorical variables. The decisions on the numerical data distribution were then made based on the mean and median comparison, standard deviation values in relation to its mean, the skewness level, kurtosis values, the Kolmogorov-Smirnov and Saphiro-Wilk normality tests, histogram of a normal curve and those of box-whisker plots. The variables with a normal distribution would be described by its mean and standard deviation (SD), while the median and inter-quartile range (IQR) would be used to depict those with a skewed distribution.

Comparison of characteristics between neonates with positive and negative blood culture was made using independent sample *t*-test for numerical variables (age of developing sepsis and body weight), and χ^2^ test or Fisher exact test for categorical variables (gender, race, onset of suspected sepsis and gestational age at birth). Comparison of PCT level between neonates with the onset of sepsis < 48 h of life and neonates with the onset of sepsis ≥ 48 h of life was made using Mann Whitney U-test. Comparison was made at three-time level, namely at initial evaluation, at 12 h and at 24 h. Similar comparison was made between neonates with positive and negative blood culture.

With blood culture results as the gold standard, diagnostic performance [sensitivity, specificity, positive (PPV) and negative predictive values (NPV)] of PCT with its 95% confidence interval were calculated separately for samples taken at 0 h, 12 h and 24 h. Discriminant validity of PCT at all three time points was assessed using the area under the Receiver Operating Characteristics (ROC) curve (AUC). An AUC of 1.0 was interpreted as a perfect score, excellent score for AUC between 0.9 and 0.99, good score for AUC between 0.8 and 0.89, fair score for AUC between 0.7 and 0.79, poor score for AUC between 0.51 and 0.69, and failing score if the AUC value is < 0.50.

This study had been granted with an ethical approval from the Human Research Ethic Committee (HREC) of USM and had received permission from the Director of HUSM to conduct the said research. Verbal and written consents from the parents or legal guardians of all the neonates who had fulfilled the study criteria were also acquired prior to the initial assessment as well as before the collection of blood samples at 0 h, 12 h and 24 h of after initial assessment. This study had also ensured that all the ethical standards that were used had complied with the principles enunciated in the Declaration of Helsinki.

## Results

The characteristics of the neonates that had been included in this study as illustrated in Table 1 had shown 12 (20%) neonates with positive blood cultures. Despite demonstrating a statistically significantly younger age (123.5 versus 65.08 h of life, *P* = 0.007) than those with negative blood cultures, the comparison of the culture results and the sepsis onset (< 48 h and ≥ 48 h of life) had however, revealed no significant difference (*P* = 0.151) between the two groups. Although the neonates with the positive blood cultures were found to have significantly lighter body weights as compared to those with negative blood cultures (1.7 kg versus 2.33 kg, *P* = 0.034), there had been no difference shown between the gender, race and birth gestational age at birth between the neonates of both blood cultures (Table 2).

The comparison of PCT level by the age of the sepsis onset had also demonstrated no significant difference at the initial time of presentation (0 h) as well as 12 h and 24 h (Table 3). As shown in Table 4, the PCT levels were found to have been consistently higher among the neonates with positive blood cultures with peaked levels at 12 h regardless of the evaluation time.

**Figure 1:**
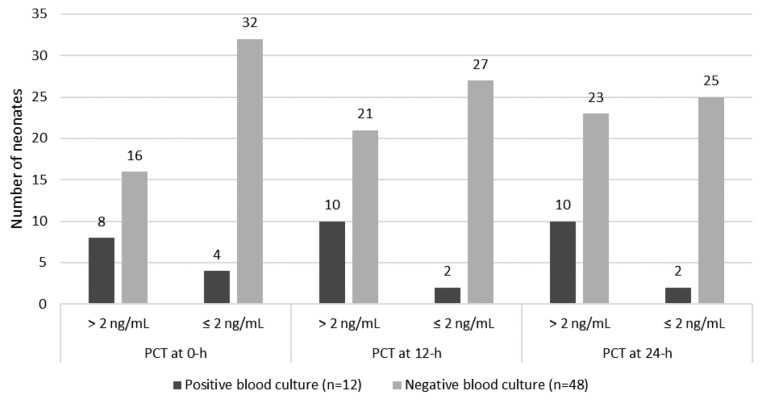
Number of neonates according to PCT and blood culture results

The number of neonates from the PCT and blood culture results at 0 h, 12 h and 24 h are shown in Figure 1, where at 0 h, 33.3% (8 out of 24) of the neonates with a PCT level of > 2 ng/mL were found to have positive blood cultures (true positive) as compared to 11.1% (4 out of 36) of those with PCT ≤ 2 ng/mL (false positive) (*P* = 0.035). A similar difference of proportion was also observed at 12 h and 24 h (32.2% versus 6.9%, *P* = 0.014 and 30.3% versus 7.4%, *P* = 0.0274).

**Figure 2:**
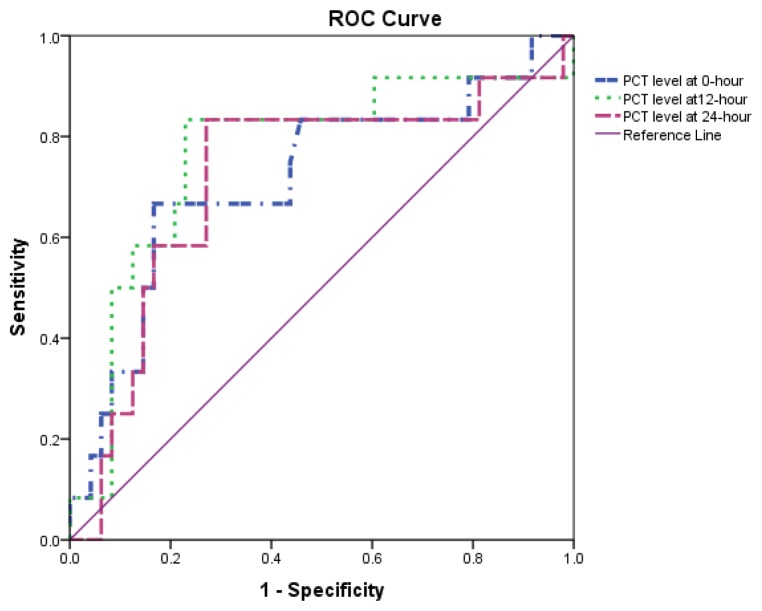
Comparison of ROC curve between PCT at 0 h, 12 h and 24 h based on a cut-off point of > 2 ng/mL for diagnosis of neonatal sepsis

By using a PCT cut-off value of > 2 ng/mL for the diagnosis of neonatal sepsis, the lowest PCT diagnostic performance was observed to have occurred at initial presentation (0 h), while the optimal performance was found to have taken place at 12 h (Table 5). Likewise, the PCT discrimination values at 0 h were also discovered to have been the lowest (AUC = 71.6%, 95% CI: 53.8%, 89.4%), which was followed by the values depicted by 24 h (AUC = 71.7%, 95% CI: 53.8%, 89.6%) and 12 h (AUC = 76.6%, 95% CI: 59.6%, 93.6%) (Figure 2).

## Discussion

The variability in the PCT diagnostic values that had been observed in various sensitivity studies with a 60% to 100% range and specificities of 79% to 100% (13) could have been influenced by the different population studies as well as the heterogeneities of the study methods (definitions of neonatal sepsis, the characteristics of the control group, the timing of blood cultures and PCT sampling as well as the cut-off points).

In this current study, low PCT sensitivity was revealed at the time of initial evaluation with improvements at the subsequent time points. It could have been affected by the presence of the low PCT level demonstrated by four out of the 12 neonates with positive blood cultures. Two of these neonates continued to have a low PCT level whilst the other two demonstrated an increased PCT level at 12 h and 24 h.

Apart from the above, the PCT levels were also found to have been significantly higher in the culture positive group at all evaluation time points as shown by their respective PCT levels of > 10 ng/mL regardless of the type of microorganisms they had been infected with.

This finding had been similar to the study conducted by Kocabas et al. (14) and Cetinkaya et al. (15), where they had respectively discovered 29 neonates with sepsis as having a significant higher PCT level than those without the illness and the serum PCT that was measured serially at 1 h–4 h and at 25 h–48 h of septic workout to be significantly higher in the septic group as compared to the non-septic group.

By using the PCT kinetics of critically ill adults as a reference point, an early blood sampling is then postulated to be one of the causes for the normal or low PCT levels in neonates with infection; since the level will only begin to rise after 2 h–4 h after the induction of an infection (16, 17). For this reason, blood samples that had been obtained too early in neonates may result in normal or low PCT levels as the infection had yet to be released into the blood system. This would thus explain the extremely low PCT levels that had been exhibited by the four neonates in this current study, where the sampling was done just less than three hours of presentation. Meanwhile, the positive blood cultures of the other two neonates with low PCT levels were found to have contained the coagulase-negative Staphylococci (CONS) with one of the affected neonates demonstrating a normal range of the total WBC and CRP. By taking both of the clinical and laboratory findings into account, it was discovered that the isolated CONS of the said subject could have been affected by the contaminated blood samples (18, 19).

The lack of specificity at all evaluation time in this current study can be partially explained by the significantly higher number of neonates with negative blood cultures as having elevated PCT levels (false positive), which had a range of between 33.3% and 47.9%. False positive PCT levels of more than 10 ng/mL had been observed in 15 neonates with nine of them having respiratory distress syndrome (RDS) caused by meconium aspiration, birth asphyxia or persistent pulmonary hypertension in newborn. There had, however, been no common risk factors identified among the rest of these neonates. As such, the elevated PCT levels in subjects with RDS as observed in our study had thus supported a previous study as reported by Gonzales et al. (20).

The high numbers of false positive PCT results in this study (based on a cut-off point of > 2 ng/mL) could also be partly explained by the normal progressive rise of the PCT during the first 48 h of after birth. In healthy neonates who were born < than 48 h, the PCT levels were seen to have changed on an hourly basis and peaked on the first day before declining to those of an adult’s after 40 h of life. This then reflects the normal physiological mechanism in neonates, which had occurred even without any infection stimulus or from other abnormal clinical starts (21). Undetected bacteraemia could also be another reason for the false positive PCT results. Since the positive blood cultures were only reported in 5%–10% of the suspected sepsis cases even at highly resourced facilities (22), these poor diagnostic performance of blood cultures could have been caused by the inadequate blood volume that were used for the cultures, the intermittent seeding of low number of bacteria within the blood stream, the increased use of prophylactic antibiotic during intra-partum periods, the timing of blood sample drawing as well as those of the blood culture systems (17, 23–25).

Diagnostic reliability or the discriminating PCT values are also influenced by the evaluation timing (26). Based on the results that were obtained from this study, the optimal sensitivity and the NPV that were achieved at 12 h rather than the 0 h and 24 h could be considered as a discriminating factor between the septic and non-septic neonates. As such, the results from our clinical setting had shown the best time point for PCT testing in neonates with suspected sepsis to be at 12 h after presentation.

One limitation of this study is that the exact onset of sepsis is unknown and all neonates were assumed to have presented immediately after the onset of sepsis. Another limitation is that the PCT had not been compared with the other infection markers such as CRP, interleukin or leucocyte count (14, 27, 28). The reliability of blood culture as a gold standard for diagnosis of sepsis has been a subject of debate, therefore the diagnostic performance of the PCT is compromised. It may be possible that some cases of true sepsis cannot be confirmed due to the false negative culture result (29, 30). The sample size of this study, which had been relatively smaller than those of the other studies (14, 31–34) could also be seen as another limitation of this current study.

## Conclusion

In conclusion, the PCT has been found to be a good indicator for neonatal sepsis. Since the diagnostic accuracy of the PCT had varied based on the time of evaluation, this study had thus proven the PCT taken at 12 h of presentation to be the optimal diagnostic value of the neonatal sepsis.

## Figures and Tables

**Table 1:** Characteristics of neonates participating in the study (*n* = 60)

Variables	Mean (SD)	*n* (%)
Age of developing sepsis, hours of life	76.8 (48.25)	
Body weight (kg)	2.25 (0.92)	
Gender		
Female		27 (45.0)
Male		33 (55.0)
Race		
Malay		57 (95.0)
Non-Malay		3 (5.0)
Onset of suspected sepsis		
< 48 h of life		17 (28.3)
≥48 h of life		43 (71.7)
Gestational age at birth (weeks)	33.9 (4.64)	
Term		25 (41.7)
Preterm		35 (58.3)

**Table 2:** Comparison of characteristics between neonates with positive and negative blood culture (*n* = 60)

Variables	Positive blood culture (*n* = 12)	Negative blood culture (*n* = 48)	Test-statistics (df)	*P*-value
Age of developing sepsis, hours of life	123.5 (60.84)	65.08 (36.86)	3.18 (13)	0.007^a^
Body weight (kg)	1.70 (0.68)	2.33 (0.94)	−2.18 (58)	0.034^a^
Gender				
Female	4 (33.3)	23 (47.9)	0.83 (1)	0.519^b^
Male	8 (66.7)	25 (52.1)		
Race				
Malay	12 (100.0)	45 (93.8)	–	>0.95^c^
Non-Malay	0 (0.0)	3 (6.3)		
Onset of suspected sepsis				
< 48h of life	1 (8.3)	16 (33.3)	–	0.151^c^
≥48h of life	11 (91.7)	21 (66.7)		
Gestational age at birth				
Term	4 (33.3)	21 (43.8)	0.43 (1)	0.513^b^
Preterm	8 (66.7)	27 (56.3)		

a Independent sample *t*-test

b χ^2^-test

c Fisher exact test

**Table 3:** Comparison of PCT levels based on onset of sepsis

PCT Level (ng/mL)	Onset < 48 h of life	Onset ≥ 48 h of life	Z	*P-*value*

	Median (IQR)			
At initial evaluation (0 hour)	0.69 (9.32)	1.32 (10.86)	−0.97	0.333
At 12 h	1.74 (25.42)	2.87 (24.54)	−0.17	0.863
At 24 h	2.20 (17.29)	2.34 (23.79)	−0.08	0.935

* Mann Whitney U-test

**Table 4:** PCT levels at different assessmenttimes(*n* = 60)

PCT Level (ng/mL)	Positive blood culture (*n* = 12)	Negative blood culture (*n* = 48)	Z	*P-*value*

	Median (IQR)			
At initial evaluation (0 hour)	11.5 (33.36)	0.9 (3.35)	−2.30	0.021
At 12-hour	32.8 (26.90)	1.5 (11.54)	−2.83	0.005
At 24-hour	24.3 (47.61)	1.7 (13.86)	−2.31	0.021

* Mann Whitney U-test

**Table 5:** Diagnostic performance of PCT at different assessment times based on a cut-off point of >2 ng/mL for diagnosis of neonatal sepsis (*n* = 60)

Time of PCT measurement	Sensitivity (%) (95% CI)	Specificity (%) (95% CI)	PPV (%) (95% CI)	NPV (%) (95% CI)
0 h	66.7 (40.0, 93.3)	66.7 (53.3, 80.0)	33.3 (14.5, 52.2)	88.9 (78.6, 99.2)
12 h	83.3 (62.2, 104.4)	56.3 (42.2, 70.3)	32.3 (15.8, 48.7)	93.1 (83.9, 102.3)
24 h	83.3 (62.2, 104.4)	52.1% (38.0, 66.2)	30.3 (14.6, 46)	92.6 (82.7, 102.5)

## References

[1] Camacho-Gonzalez A, Spearman PW, Stoll BJ (2013). Neonatal infectious diseases: evaluation of neonatal sepsis. Pediatr Clin North Am.

[2] Shah BA, Padbury JF (2014). Neonatal sepsis: an old problem with new insights. Virulence.

[3] Qazi SA, Stoll BJ (2009). Neonatal sepsis: a major global public health challenge. Pediatr Infect Dis J.

[4] Faix JD (2013). Biomarkers of sepsis. Crit Rev Clin Lab Sci.

[5] Arnon S, Litmanovitz I (2008). Diagnostic tests in neonatal sepsis. Curr Opin Infect Dis.

[6] Tappero E, Johnson P (2010). Laboratory evaluation of neonatal sepsis. Newborn Infant Nurs Rev.

[7] Palmiere C, Mangin P (2012). Postmortem chemistry update part II. Int J Legal Med.

[8] Bréchot N, Hékimian G, Chastre J, Luyt C-E (2015). Procalcitonin to guide antibiotic therapy in the ICU. Int J Antimicrob Agents.

[9] Adib M, Bakhshiani Z, Navaei F, Saheb Fosoul F, Fouladi S, Kazemzadeh H (2012). Procalcitonin: a reliable marker for the diagnosis of neonatal sepsis. Iran J Basic Med Sci.

[10] Khoshdel A, Mahmoudzadeh M, Kheiri S, Imani R, Shahabi G, Saedi E (2008). Sensitivity and specificity of procalcitonin in diagnosis of neonatal sepsis. Iranian Journal of Pathology.

[11] Tiskumara R, Fakharee SH, Liu CQ, Nuntnarumit P, Lui KM, Hammoud M (2009). Neonatal infections in Asia. Arch Dis Child Fetal Neonatal Ed.

[12] Vouloumanou EK, Plessa E, Karageorgopoulos DE, Mantadakis E, Falagas ME (2011). Serum procalcitonin as a diagnostic marker for neonatal sepsis: a systematic review and meta-analysis. Intensive Care Medicine.

[13] Aboud MI, Waise MMA, Shakerdi LA (2010). Procalcitonin as a marker of neonatal sepsis in intensive care units. Iranian Journal of Medical Sciences.

[14] Kocabas E, Sarikcioglu A, Aksaray N, Seydaoglu G, Seyhun Y, Yaman A (2007). Role of procalcitonin, C-reactive protein, interleukin-6, interleukin-8 and tumor necrosis factor-alpha in the diagnosis of neonatal sepsis. Turk J Pediatr.

[15] Cetinkaya M, Özkan H, Köksal N, Celebi S, Hacımustafaoğlu M (2009). Comparison of serum amyloid A concentrations with those of C-reactive protein and procalcitonin in diagnosis and follow-up of neonatal sepsis in premature infants. J Perinatol.

[16] Meisner M (2014). Update on procalcitonin measurements. Ann Lab Med.

[17] Chan T, Gu F (2011). Early diagnosis of sepsis using serum biomarkers. Expert Rev Mol Diagn.

[18] Loonen AJ, de Jager CP, Tosserams J, Kusters R, Hilbink M, Wever PC (2014). Biomarkers and molecular analysis to improve bloodstream infection diagnostics in an emergency care unit. PLoS One.

[19] Healy CM, Baker CJ, Palazzi DL, Campbell JR, Edwards MS (2013). Distinguishing true coagulase-negative Staphylococcus infections from contaminants in the neonatal intensive care unit. J Perinatol.

[20] Gonzales JN, Lucas R, Verin AD (2015). The acute respiratory distress syndrome: mechanisms and perspective therapeutic approaches. Austin J Vasc Med.

[21] Davidson J, Tong S, Hauck A, Lawson DS, da Cruz E, Kaufman J (2013). Kinetics of procalcitonin and C-reactive protein and the relationship to postoperative infection in young infants undergoing cardiovascular surgery. Pediatr Res.

[22] Edmond K, Zaidi A (2010). New approaches to preventing, diagnosing, and treating neonatal sepsis. PLoS Med.

[23] Su H, Chang S, Han C, Wu K, Li M, Huang C (2014). Inflammatory markers in cord blood or maternal serum for early detection of neonatal sepsis—a systemic review and meta-analysis. J Perinatol.

[24] Lin HH, Liu YF, Tien N, Ho CM, Hsu LN, Lu JJ (2013). Evaluation of the blood volume effect on the diagnosis of bacteremia in automated blood culture systems. J Microbiol Immunol Infect.

[25] Opota O, Jaton K, Greub G (2015). Microbial diagnosis of bloodstream infection: towards molecular diagnosis directly from blood. Clin Microbiol Infect.

[26] Altunhan H, Annagür A, Örs R, Mehmetoğlu I (2011). Procalcitonin measurement at 24 hours of age may be helpful in the prompt diagnosis of early-onset neonatal sepsis. Int J Infect Dis.

[27] Park IH, Lee SH, Yu ST, Oh YK (2014). Serum procalcitonin as a diagnostic marker of neonatal sepsis. Korean Journal of Pediatrics.

[28] Hasan F, Khan SA, Maharoof MK, Muhammed N (2017). Role of procalcitonin in early diagnosis of neonatal sepsis. International Journal of Contemporary Pediatrics.

[29] Klingenberg C, Kornelisse RF, Buonocore G, Maier RF, Stocker M (2018). Culture-negative early-onset neonatal sepsis-at the crossroad between efficient sepsis care and antimicrobial stewardship. Front Pediatr.

[30] Lin EC, Boehm KM (2013). Positive predictive value of blood cultures utilized by community emergency physicians. ISRN Infectious Diseases.

[31] Sastre JBL, Solís DP, Serradilla VR, Colomer BF, Cotallo GDCJB (2007). Evaluation of procalcitonin for diagnosis of neonatal sepsis of vertical transmission. BMC Pediatr.

[32] Chiesa C, Panero A, Rossi N, Stegagno M, De Giusti M, Osborn JF (1998). Reliability of procalcitonin concentrations for the diagnosis of sepsis in critically ill neonates. Clin Infect Dis.

[33] Gendrel D, Assicot M, Raymond J, Moulin F, Francoual C, Badoual J (1996). Procalcitonin as a marker for the early diagnosis of neonatal infection. J Pediatr.

[34] Guibourdenche J, Bedu A, Petzold L, Marchand M, Mariani-Kurdjian P, Hurtaud-Roux MF (2002). Biochemical markers of neonatal sepsis: value of procalcitonin in the emergency setting. Annals of Clinical Biochemistry.

